# Glucocorticoid receptor inhibits Müller glial galectin‐1 expression via DUSP1‐dependent and ‐independent deactivation of AP‐1 signalling

**DOI:** 10.1111/jcmm.14559

**Published:** 2019-07-21

**Authors:** Ikuyo Hirose, Atsuhiro Kanda, Kousuke Noda, Susumu Ishida

**Affiliations:** ^1^ Laboratory of Ocular Cell Biology and Visual Science, Department of Ophthalmology, Faculty of Medicine and Graduate School of Medicine Hokkaido University Sapporo Japan

**Keywords:** activator protein‐1, diabetic retinopathy, dual specificity phosphatase 1, galectin‐1, glucocorticoid receptor, interleukin‐1β, Müller glia, transactivation, transrepression

## Abstract

Galectin‐1/*LGALS1* is a hypoxia‐induced angiogenic factor associated with diabetic retinopathy (DR). Recently, we elucidated a hypoxia‐independent pathway to produce galectin‐1 in Müller glial cells stimulated by interleukin (IL)‐1β. Here we revealed glucocorticoid receptor (GR)‐mediated inhibitory mechanisms for Müller glial galectin‐1/*LGALS1* expression. Activator protein (AP)‐1 site in the *LGALS1* enhancer region, to which activating transcription factor2, c‐Fos and c‐Jun bind, was shown to be essential for IL‐1β‐induced galectin‐1/*LGALS1* expression in Müller cells. Ligand (dexamethasone or triamcinolone acetonide)‐activated GR induced dual specificity phosphatase (DUSP)1 expression via the glucocorticoid response element and attenuated IL‐1β‐induced galectin‐1/*LGALS1* expression by reducing phosphorylation of these AP‐1 subunits following AKT and extracellular signal‐regulated kinase (ERK)1/2 deactivation. Moreover, activated GR also caused DUSP1‐independent down‐regulation of IL‐1β‐induced *LGALS1* expression via its binding to AP‐1. Administration of glucocorticoids to mice attenuated diabetes‐induced retinal galectin‐1/*Lgals1* expression together with AKT/AP‐1 and ERK/AP‐1 pathways. Supporting these in vitro and in vivo findings, immunofluorescence analyses showed co‐localization of galectin‐1 with GR and phosphorylated AP‐1 in DUSP1‐positive glial cells in fibrovascular tissues from patients with DR. Our present data demonstrated the inhibitory effects of glucocorticoids on glial galectin‐1 expression via DUSP1‐dependent and ‐independent deactivation of AP‐1 signalling (transactivation and transrepression), highlighting therapeutic implications for DR.

## 1 INTRODUCTION

Galectin‐1, encoded by *lectin, galactoside‐binding, soluble* (*LGALS*)*1* gene, is a lectin family protein that recognizes galactose‐containing sugar chains and exerts various biological actions through its binding to the carbohydrate‐coated proteins.^1^ Recently, galectin‐1 was shown to be an agonistic ligand for vascular endothelial growth factor (VEGF) receptor (VEGFR)2 via its lectin activity and a hypoxia‐induced angiogenic factor related to cancer progression^2^ as well as retinal neovascularization in rodents.^3^ Our human sample data showed the significant association of galectin‐1 with the neovascular pathogenesis of diabetic retinopathy (DR).^4^ Müller glial cells were suggested as a cellular source of its production in the diabetic retina.^4^ As there was no correlation between VEGF and galectin‐1 protein levels in the eye,^4^ VEGF‐unrelated regulatory pathways were speculated to exist to induce galectin‐1. Indeed, we also reported that galectin‐1 levels increased in DR eyes along with the progression of clinical stages from the pre‐ischaemic (background) stage with macular oedema.^5^ As concerns the hypoxia‐independent mechanism of galectin‐1 expression, we revealed that advanced glycation end product (AGE)‐induced interleukin (IL)‐1β activated extracellular signal‐regulated kinase (ERK)1/2 and phosphatidylinositol‐3 kinase (PI3K)/AKT pathways in Müller glial cells, leading to galectin‐1 expression via these diabetes‐associated inflammatory cascades.^5^ Importantly, IL‐1β was expressed in AGE‐positive macrophages migrating into the fibrovascular tissue from proliferative DR patients, and in vivo macrophage depletion significantly reduced diabetes‐induced retinal *Il1b* and *Lgals1* expression levels.^5^

As the pathogenesis of DR harbours AGE‐driven chronic inflammation, glucocorticoid drugs including dexamethasone and triamcinolone acetonide are currently used for the treatment of diabetic macular oedema as an anti‐inflammatory strategy, in addition to the first‐line anti‐VEGF therapy. On top of its pro‐angiogenic function, VEGF, originally reported as vascular permeability factor,^6^ is also regarded as an inflammatory cytokine that causes oedematous and exudative lesions. Nowadays, the anti‐VEGF drugs ranibizumab and aflibercept have expanded their applications from diabetic macular oedema to proliferative DR^7^, ^8^ and are established as a gold standard therapeutic strategy broadly covering the early to late clinical stages of DR. However, DR is a complex disorder associated with multiple molecules, and anti‐VEGF refractory cases seen in clinical practice are likely to ensue from several other candidates responsive for the pathogenesis of DR. Of these, galectin‐1, a hypoxia‐induced angiogenic factor, is theorized to be another important therapeutic target covering the entire clinical stages of DR, given that galectin‐1, as well as VEGF, contributes to the inflammatory pathogenesis of DR prior to the proliferative (angiogenic) stage.^5^ Moreover, galectin‐1 showed no correlation with VEGF in intraocular protein concentration^4^ possibly because of its different induction pathway caused by AGE‐driven inflammation rather than hypoxia,^5^ highlighting its potential as an alternative and complementary target in the treatment of anti‐VEGF refractory cases. Detailed investigation into mechanisms for inducing the expression of and blocking the function of galectin‐1 would therefore be highly warranted in order to improve the long‐term management of DR.

Aflibercept is a chimeric glycoprotein consisting of the VEGF‐binding domains of VEGFR1 and VEGFR2 fused to the Fc portion of immunoglobulin (Ig)G.^9^ Previously, we reported the neutralizing efficacy of aflibercept against galectin‐1, which utilizes the binding affinity between sugar chains on the VEGFR2 portion and galectin‐1.^4^ On top of its blockade by aflibercept, we herein revealed a novel mechanism related to galectin‐1 down‐regulation, in which glucocorticoids inhibit IL‐1β‐induced galectin‐1 expression in Müller glial cells via transactivation and transrepression. These findings were further supported by blocking experiments with diabetic animals and tissue localization of related proteins in human DR samples.

## 2 MATERIALS AND METHODS

### 2.1 Cell line and reagents

The human Müller glial cell line Moorfields/Institute of Ophthalmology‐Müller 1 (MIO‐M1) was provided from Dr G. Astrid Limb (UCL Institute of Ophthalmology).^10^ The cells were cultured in DMEM containing 10% FBS (Thermo Fisher Scientific). Twenty‐four hours after transfection, the composite transfection mixture was removed and replaced with 1% FBS/DMEM for 24 hours, followed with recombinant protein and reagent treatments before each assay. Recombinant human IL‐1β proteins were purchased from R&D systems. Aldosterone and streptozotocin (STZ) were from Sigma‐Aldrich. RU486 was from Cayman Chemical. Dexamethasone sodium phosphate, triamcinolone acetonide and LY294002 (PI3K inhibitor) were from FUJIFILM Wako Pure Chemical Corporation. U0126 (ERK1/2 inhibitor) was from Promega. Reagents were dissolved in either PBS, ethanol (final concentration < 0.1%) or dimethyl sulfoxide (DMSO, final concentration < 0.1%).

Specific siRNAs against *dual specificity phosphatase* (*DUSP*)*1* (hs.Ri.DUSP1.13.3), *TSC22 domain family member* (*TSC22D*)*3* siRNA (hs.Ri.TSC22D3.13.1) and a negative control siRNA oligo (DS NC1) were purchased from Integrated DNA Technologies and used at 10 nmol/L. Cells were transfected with siRNA using Lipofectamine RNAiMAX Reagent (Thermo Fisher Scientific) following the manufacturer's protocols.

### 2.2 Reporter assays

To generate *LGALS1* promoter‐enhancer reporter constructs,^11^, ^12^, ^13^ three fragments spanning nucleotides −500 bp to +67 bp from the *LGALS1* transcription start site (promoter region), +450 bp to +1750 bp (enhancer region), and AP‐1 site (TGACTCA)‐mutated enhancer region were synthesized and sequenced by Integrated DNA Technologies, and subcloned into the pGL4 promoterless reporter vector (Promega), generating pGal, pGal + AP‐1 and pGalΔAP‐1. The pRL‐CMV Renilla luciferase plasmid (Promega) was used as internal control. The Dual‐Luciferase Reporter Assays System (Promega) was used to measure the activity of firefly and Renilla luciferase. Cells were transfected with plasmid DNA using Lipofectamine LTX with Plus Reagent (Thermo Fisher Scientific) following the manufacturer's protocols.

The Pathway Profiling SEAP System (TAKARA BIO) was utilized to detect pathways in response to corticosteroids following the manufacturer's recommendations. After transfection, the medium was collected, and secreted alkaline phosphatase was measured using the chemiluminescent alkaline phosphatase detection kit Great EscAPe™ SEAP (TAKARA BIO) according to the manufacturer's procedures.

### 2.3 Chromatin immunoprecipitation‐quantitative PCR (ChIP‐qPCR)

Assays were performed using the SimpleChIP Enzymatic Immunoprecipitation Chromatin IP Kit (Cell Signaling Technology) according to the manufacturer's protocols. Chromatin was immunoprecipitated with rabbit antibodies against activating transcription factor (ATF)2, c‐Fos, FosB, c‐Jun (Cell Signaling Technology) and glucocorticoid receptor (GR) (Thermo Fisher Scientific). Normal rabbit IgG (Cell Signaling Technology) was used as control. Thereafter, chromatin immunoprecipitates were evaluated by real‐time quantitative PCR (qPCR) using the primers specific for the previously described AP‐1‐binding site in the *LGALS1* enhancer region^13^ and GR‐binding site in the *DUSP1* promoter region,^14^ together with 2% input DNA as reference samples. All primers are listed in Table S1. Real‐time qPCR was performed using KOD SYBR qPCR Mix (TOYOBO, Tokyo, Japan) and StepOne Plus Systems (Thermo Fisher Scientific). ChIP‐qPCR signals were calculated as percentage of input.

### 2.4 Immunoblot analyses

Cell extracts were lysed in SDS buffer, a protease inhibitor cocktail (Promega) and phosphatase inhibitor cocktail (FUJIFILM Wako Pure Chemical Corporation). After quantifying protein concentrations using BCA reagent (Thermo Fisher Scientific), proteins were resolved by SDS‐PAGE and transferred to nitrocellulose membrane by electroblotting. Membranes were blocked in TBS containing 5% skim milk and probed with the following primary antibodies: goat anti‐galectin‐1 antibody (1:1000, R&D systems), rabbit antibodies against ATF2 (1:1000), phosphorylated ATF2 (1:1000), c‐Fos, phosphorylated c‐Fos (1:1000), c‐Jun (1:1000), phosphorylated c‐Jun (1:1000), AKT (also known as protein kinase B) (1:1000), phosphorylated AKT (1:1000), ERK1/2 (1:1000), phosphorylated ERK1/2 (1:1000, Cell Signaling Technology), DUSP1 (1:1000, Millipore) and β‐actin (1:4000, Medical & Biological Laboratories). Horseradish peroxidase‐conjugated anti‐goat and anti‐rabbit IgGs (1:4000, Jackson ImmunoResearch Laboratories) were used as a secondary antibody for chemoluminescence detection. Signals were obtained by enhanced chemoluminescence (Perkin Elmer).

### 2.5 Human surgical samples

During surgery (vitrectomy for traction retinal detachment), five fibrovascular tissues were excised from eyes of patients with proliferative DR and used for immunohistochemistry. This study was conducted in accordance with the tenets of the Declaration of Helsinki and after receiving approval from the institutional review board of Hokkaido University Hospital. Written informed consent was obtained from all patients after an explanation of the purpose and procedures of this study.

### 2.6 Immunofluorescence microscopy

Immunofluorescence analyses were performed as described previously.^4^, ^5^, ^15^ Serial sections were incubated with the following primary antibodies: mouse anti‐galectin‐1 (1:50, Santa Cruz Biotechnology), mouse and rabbit anti‐glial fibrillary acidic protein (GFAP) (1:200, Leica), rabbit anti‐phosphorylated ATF2 (1:100), rabbit anti‐phosphorylated c‐Fos (1:100), and rabbit anti‐phosphorylated c‐Jun (1:100, Cell Signaling Technology), rabbit anti‐DUSP1 (1:100, Millipore) and rabbit anti‐GR (1:100, Thermo Fisher Scientific) antibodies. Secondary antibodies for fluorescent detection were AlexaFluor 488 and 546 (1:500, Thermo Fisher Scientific). Nuclei were counterstained with DAPI, and sections were visualized under a Keyence BZ‐9000 (Keyence).

### 2.7 Real‐time quantitative PCR (qPCR)

Total RNA isolation and reverse transcription were performed from cells using SuperPrep Cell Lysis & RT Kit for qPCR (TOYOBO) and from tissues using PureLink RNA Mini Kit (Thermo Fisher Scientifc) and GoScrip Reverse Transcriptase (Promega), as previously described.^4^, ^5^, ^15^ All primers are listed in Table S1. Real‐time qPCR was performed using the GoTaq qPCR Master Mix (Promega) and StepOne Plus Systems (Thermo Fisher Scientific). Gene expression levels were calculated using the 2^−ddCt^ method.

### 2.8 Animals and induction of diabetes

C57BL/6J mice were obtained from CLEA Japan. All animal experiments were conducted in accordance with the ARVO (Association of Research in Vision and Ophthalmology) Statement for the Use of Animals in Ophthalmic and Vision Research and approved by the Ethics Review Committee for Animal Experimentation of Hokkaido University. Procedures for murine model of STZ‐induced diabetes were described in our previous report.^4^, ^5^ At 2 months after induction of diabetes, dexamethasone and triamcinolone acetonide (50 pmol/eye for each) were injected into the vitreous cavity.

### 2.9 Statistical analyses

All the results are expressed as the mean ± SEM (standard error of the mean). Student's *t* test was used for statistical comparison between groups, and one‐way analysis of variance (ANOVA) followed by the Tukey‐Kramer method as a post hoc test was used for multiple comparison procedures. Differences between means were considered statistically significant when *P* values were <.05.

## 3 RESULTS

### 3.1 Requirement of AKT‐ and ERK1/2‐dependent AP‐1 activity in IL‐1β‐induced *LGALS1* expression in Müller glial cells

**Figure 1:**
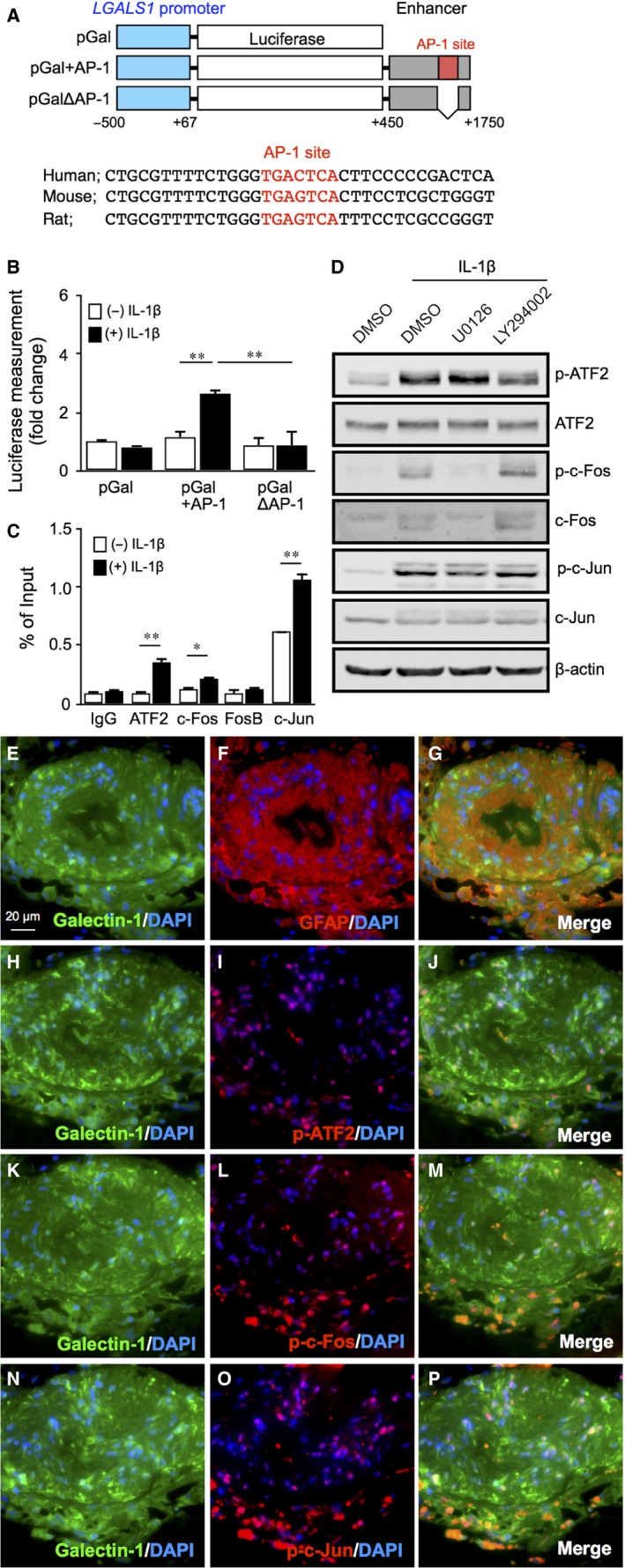
Requirement of AKT‐ and ERK1/2‐dependent AP‐1 activity in IL‐1β‐induced *LGALS1* expression in Müller glial cells. A, Upper, schematic representation of *LGALS1* promoter‐ and enhancer‐driven luciferase constructs. Lower, sequence comparison between human, mouse and rat *LGALS1* enhancer regions spanning AP‐1 site (*red*). B, AP‐1 site in the *LGALS1* enhancer region was required for IL‐1β‐enhanced *LGALS1* promoter‐driven luciferase activity. Transfected Müller glial cells were stimulated with IL‐1β (10 ng/mL) for 24 h and assayed for luciferase activity. C, Müller glial cells were stimulated with IL‐1β (10 ng/mL) for 1 h before harvest of samples. Binding of ATF2, c‐Fos, FosB and c‐Jun to AP‐1 site in the *LGALS1* enhancer region was analysed by ChIP‐qPCR. *P < .05, **P < .01, n = 4‐6 per group. D, Müller glial cells were pre‐treated with each inhibitor at 10 μmol/L for 30 min before stimulation with IL‐1β (10 ng/mL) for 1 h, and protein levels of phosphorylated and total forms of AP‐1 subunits were analysed. (E‐P) Double labelling of galectin‐1 (*green*), GFAP (*red*) and DAPI (*blue*) (E‐G); galectin‐1 (*green*), phosphorylated ATF2 (*red*) and DAPI (*blue*) (H‐J); galectin‐1 (*green*), phosphorylated c‐Fos (*red*) and DAPI (*blue*) (K‐M); galectin‐1 (*green*), phosphorylated c‐Jun (*red*) and DAPI (*blue*) (N‐P) in fibrovascular tissues excised from human eyes with proliferative DR. Scale bar = 20 μm

In addition to galectin‐1 expression regulated by hypoxia,^4^, ^5^, ^16^ we recently revealed IL‐1β‐induced *LGALS1* gene expression via PI3K/AKT and ERK1/2 mitogen‐activated protein kinase (MAPK) pathways in human Müller glial cells.^5^ To further elucidate the regulatory mechanisms of IL‐1β‐induced *LGALS1* gene expression, we analysed the promoter region of *LGALS1*. Given that the 0.5 kbp upstream region from the transcription start site of *LGALS1* promoter plays a critical role in *LGALS1* gene expression,^11^, ^12^ we first generated a luciferase vector driven by *LGALS1* promoter (−500 bp to +67 bp, pGal) (Figure 1A). However, there was no significant increase in luciferase activity after IL‐1β stimulation (Figure 1B). Previous reports showed that *LGALS1* expression in classical Hodgkin's lymphoma was mediated in part by a highly conserved (in humans and rodents) AP‐1‐dependent *LGALS1* enhancer, located in the enhancer region (+450 bp to +1750 bp)^12^, ^13^ (Figure 1A). To determine the role of AP‐1‐dependent *LGALS1* enhancer in Müller glial cells, we next studied whether *LGALS1* enhancer is selectively active because of IL‐1β stimulation. The construct including the enhancer region (pGal + AP‐1) did not change luciferase activity without IL‐1β stimulation, as compared to the construct without the enhancer region (pGal); however, the administration of IL‐1β significantly increased luciferase activity in the enhancer‐containing construct (pGal + AP‐1). Additionally, we observed that the construct lacking AP‐1 site (+1594 bp to +1600 bp, pGalΔAP‐1) exhibited significantly lower luciferase activity (Figure 1B), suggesting that AP‐1 site in the *LGALS1* enhancer region is essential for IL‐1β‐induced galectin‐1/*LGALS1* expression in Müller glial cells.

Transcription factors including AP‐1 and nuclear factor (NF)‐κB are involved in the up‐regulation of inflammatory gene expression. As AP‐1 subunit c‐Jun was shown to up‐regulate *LGALS1* expression through AP‐1 site in lymphoma cells,^12^, ^13^ we examined the involvement of AP‐1, mainly composed of ATF, Fos and Jun family protein dimers, in IL‐1β‐stimulated *LGALS1* expression in Müller glial cells. ChIP‐qPCR revealed that binding of ATF2, c‐Fos and c‐Jun, but not FosB, to AP‐1 site in the *LGALS1* enhancer region significantly increased after stimulation with IL‐1β (Figure 1C). Supporting these findings, administration of IL‐1β to Müller glial cells increased the phosphorylated levels of ATF2, c‐Fos and c‐Jun, all of which were reduced by inhibition of ERK1/2 MAPK signalling with U0126 or PI3K/AKT signalling with LY294002 (Figure 1D). These results suggest the facilitatory role of AKT/AP‐1 and ERK/AP‐1 pathways in IL‐1β‐induced galectin‐1/*LGALS1* expression in Müller glial cells.

To examine the tissue localization and expression of galectin‐1 and AP‐1 subunits, we carried out immunofluorescence analyses on fibrovascular tissue samples collected from patients with proliferative DR. In serial sections of the fibrovascular tissues, galectin‐1 was immunoreactive in GFAP‐positive glial cells (Figure 1E‐G), whose nuclei were also immunopositive for phosphorylated ATF2 (Figure 1H‐J), phosphorylated c‐Fos (Figure 1K‐M) and phosphorylated c‐Jun (Figure 1N‐P), suggesting the regulatory role of AP‐1 in the glial expression of galectin‐1 in human DR.

### 3.2 **Glucocorticoid‐mediated suppression of IL‐1**β**‐induced galectin‐1/*LGALS1* expression with AKT/AP‐1 and ERK/AP‐1 activation in Müller glial cells**

**Figure 2:**
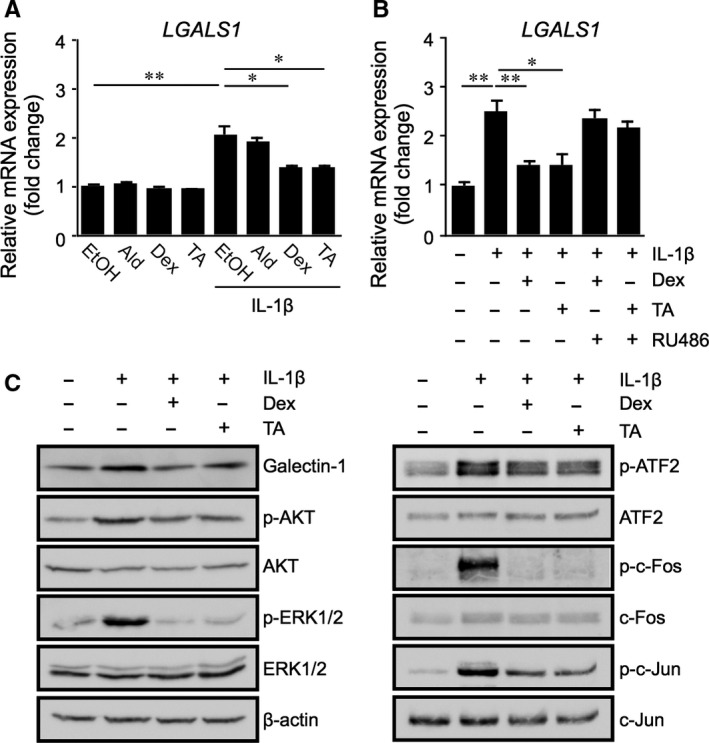
Glucocorticoid‐mediated suppression of IL‐1β‐induced galectin‐1/*LGALS1* expression with AKT/AP‐1 and ERK/AP‐1 activation in Müller glial cells. A, Müller glial cells were pre‐treated with aldosterone (Ald, 1 μmol/L), dexamethasone (Dex, 1 μmol/L) or triamcinolone acetonide (TA, 1 μmol/L) for 30 min before stimulation with IL‐1β (10 ng/mL) for 24 h, and *LGALS1* gene expression levels were analysed. B, Müller glial cells were pre‐treated with the GR antagonist (RU486, 1 μmol/L) for 30 min before application with Dex (1 μmol/L), TA (1 μmol/L) and IL‐1β (10 ng/mL) for 24 h, and *LGALS1* gene expression levels were analysed. *P < .05, **P < .01, n = 8 per group. C, Müller glial cells were pre‐treated with Dex or TA at 1 μmol/L for 30 min before stimulation with IL‐1β (10 ng/mL) for 24 h, and protein levels of galectin‐1, phosphorylated and total forms of AKT, ERK1/2 and AP‐1 subunits were analysed

Glucocorticoid drugs, such as dexamethasone and triamcinolone acetonide, bind to GR, which belongs to the nuclear receptor superfamily of ligand‐dependent transcription factors, and exert activation and repression of specific target genes. Next, we hypothesized that glucocorticoids act as a potential suppressors of IL‐1β‐induced galectin‐1/*LGALS1* expression. Although application with neither dexamethasone nor triamcinolone acetonide to Müller glial cells affected basal *LGALS1* mRNA levels, IL‐1β‐induced *LGALS1* mRNA levels were significantly reduced by these glucocorticoids, but not by the mineralocorticoid aldosterone (Figure 2A). Moreover, the suppressive effects of the glucocorticoids on *LGALS1* mRNA expression were cancelled by pre‐treatment with the GR antagonist RU486 (Figure 2B). We also confirmed that the glucocorticoids reduced IL‐1β‐induced elevation of phosphorylated AP‐1 subunits ATF2, c‐Fos and c‐Jun as well as phosphorylated AKT and ERK1/2, on top of galectin‐1 protein (Figure 2C), suggesting that glucocorticoid‐mediated suppression of IL‐1β‐induced galectin‐1/*LGALS1* expression resulted from deactivation of AKT/AP‐1 and ERK/AP‐1 pathways in Müller glial cells.

### 3.3 **Glucocorticoid‐transactivated DUSP1 expression as an exclusive suppressor of IL‐1**β**‐induced galectin‐1/*LGALS1* expression with AKT and ERK1/2 activation in Müller glial cells**

**Figure 3:**
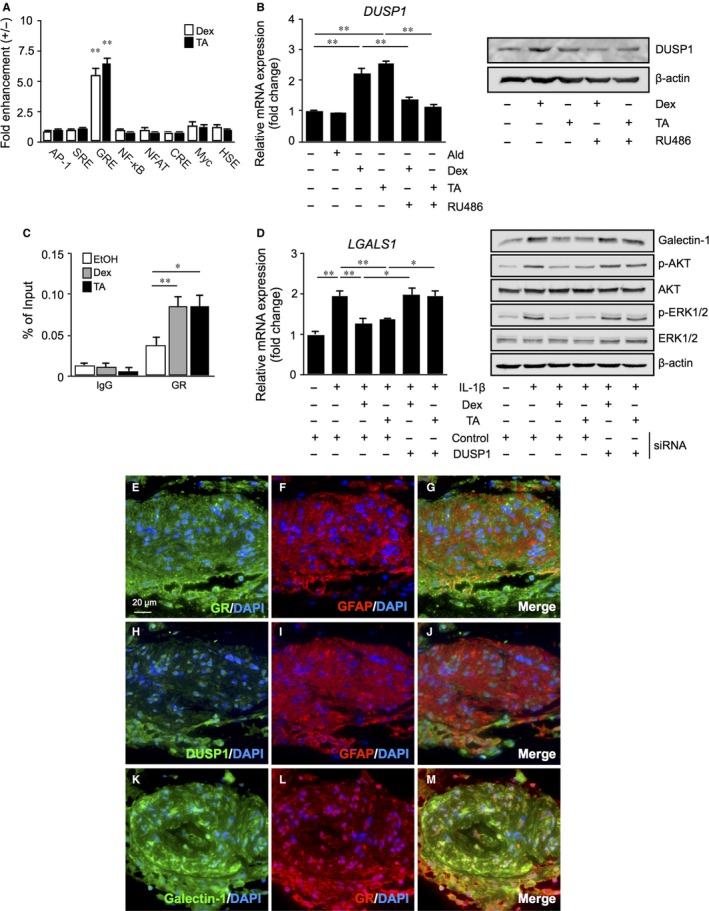
Glucocorticoid‐transactivated DUSP1 expression as an exclusive suppressor of IL‐1β‐induced galectin‐1/*LGALS1* expression with AKT and ERK1/2 activation in Müller glial cells. A, Transcriptional factors activated by glucocorticoids. Müller glial cells were transfected with plasmids containing the following response elements or binding sites (AP‐1; SRE, serum response element; GRE; NF‐κB; NFAT, nuclear factor of activated T cells; CRE, cAMP response element; Myc, E‐box DNA‐binding element; HSE, heat shock response element). After 24 h, cells were stimulated with dexamethasone (Dex, 1 μmol/L) or triamcinolone acetonide (TA, 1 μmol/L) for 48 h and assayed for alkaline phosphatase activity. Fold change is relative to vehicle‐treated controls. B, Müller glial cells were pre‐treated with the GR antagonist RU486 (1 μmol/L) for 30 min before application with aldosterone (Ald, 1 μmol/L), Dex (1 μmol/L) or TA (1 μmol/L) for 24 h, and *DUSP1* gene and protein expression levels were analysed. n = 6‐8 per group. C, Müller glial cells were treated with Dex (1 μmol/L) or TA (1 μmol/L) for 1 h, and GR binding to GRE in the *DUSP1* promoter region was analysed by ChIP‐qPCR. n = 4‐6 per group. D, After *DUSP1* knockdown, cells were applied with Dex (1 μmol/L) or TA (1 μmol/L) and 10 ng/mL IL‐1β for 24 h, *LGALS1* gene and protein expression levels were analysed. Immunoblots were probed with antibodies against galectin‐1 and phosphorylated and total forms of AKT and ERK1/2. *P < .05, **P < .01, n = 6‐8 per group. (E‐M) Double labelling of GR (*green*), GFAP (*red*) and DAPI (*blue*) (E‐G); DUSP1 (*green*), GFAP (*red*) and DAPI (*blue*) (H‐J); galectin‐1 (*green*), GR (*red*) and DAPI (*blue*) (K‐M) in fibrovascular tissues excised from human eyes with proliferative DR. Scale bar = 20 μm

To explore the repressive effect of glucocorticoids on galectin‐1 production, we performed reporter gene analyses in Müller glial cells treated with glucocorticoids using cell signalling pathway profiling systems to identify the activation of key signalling pathways. Reporter activity levels of glucocorticoid response element (GRE), but not any of the other response elements or binding sites, significantly increased after treatment with dexamethasone and triamcinolone acetonide to Müller glial cells when compared to ethanol as a vehicle control (Figure 3A), suggesting that the glucocorticoids exert their therapeutic efficacy via binding to GRE and activating its promoter function in Müller glial cells.

A number of glucocorticoid‐transactivated genes have been shown to contribute to the anti‐inflammatory actions of glucocorticoid‐GR signalling mediated via GREs.^17^ Next, to identify the molecular mechanism of deactivation of PI3K/AKT and ERK1/2 MAPK pathways in Müller glial cells treated with glucocorticoids (Figure 2C), we examined the mRNA expression of *DUSP1* (also known as MAPK phosphatase‐1, MKP‐1), *ANXA1* (Annexin A1) and *TSC22D3* (also known as glucocorticoid‐induced leucine zipper, GILZ), all of which are major anti‐inflammatory molecules that mitigate MAPK signalling pathways.^17^, ^18^ Application with dexamethasone and triamcinolone acetonide, but not aldosterone, to Müller glial cells significantly increased the expression of *DUSP1*, which was abolished by pre‐treatment with RU486 (Figure 3B). Consistently, immunoblotting demonstrated the impact of the glucocorticoids on DUSP1 protein levels as well (Figure 3B). ChIP‐qPCR revealed that GR binding to GRE in the *DUSP1* promoter region significantly increased after stimulation with dexamethasone and triamcinolone acetonide (Figure 3C), in accordance with glucocorticoid‐induced GRE reporter activity (Figure 3A) and DUSP1 expression (Figure 3B).

To confirm the involvement of DUSP1 in the suppression of IL‐1β‐induced galectin‐1/*LGALS1* expression, *DUSP1* mRNA was knocked down using siRNA in Müller glial cells. Preliminary results confirmed the potent inhibition of glucocorticoid‐activated *DUSP1* mRNA and protein expression levels by siRNA (Figure S1A). Interestingly, the siRNA‐based depletion of *DUSP1* mRNA reversed glucocorticoid‐mediated down‐regulation of *LGALS1* transcript (Figure 3D). Supporting the gene expression data, immunoblotting also showed that *DUSP1* knockdown cancelled glucocorticoid‐mediated suppression of galectin‐1 production with AKT and ERK1/2 phosphorylation in Müller glial cells (Figure 3D).

Furthermore, we investigated whether *ANXA1* and *TSC22D3* contribute to the molecular mechanism of glucocorticoid‐mediated galectin‐1/*LGALS1* down‐regulation. Administration with dexamethasone and triamcinolone acetonide to Müller glial cells significantly increased the expression of *TSC22D3*, but not *ANXA1* (Figure S1B,C). However, siRNA‐based silencing of *TSC22D3* did not cancel glucocorticoid‐mediated suppression of IL‐1β‐induced galectin‐1/*LGALS1* expression (Figure S1D,E). These results suggested that glucocorticoid‐transactivated DUSP1 exclusively suppressed IL‐1β‐activated galectin‐1/*LGALS1* expression in Müller glial cells.

Next, we carried out immunofluorescence for GR using serial sections of the surgically excised fibrovascular tissues from patients with proliferative DR. GFAP‐positive glial cells expressed GR (Figure 3E‐G) and DUSP1 (Figure 3H‐J) in the fibrovascular tissues, in which galectin‐1 colocalized with GR (Figure 3K‐M), suggesting that glial cells have a capacity for regulating the expression of galectin‐1 via DUSP1 in response to glucocorticoids in the treatment of DR.

### 3.4 DUSP1‐independent down‐regulation of *LGALS1* expression because of deactivation of AP‐1 signalling via GR binding to AP‐1 in Müller glial cells

**Figure 4:**
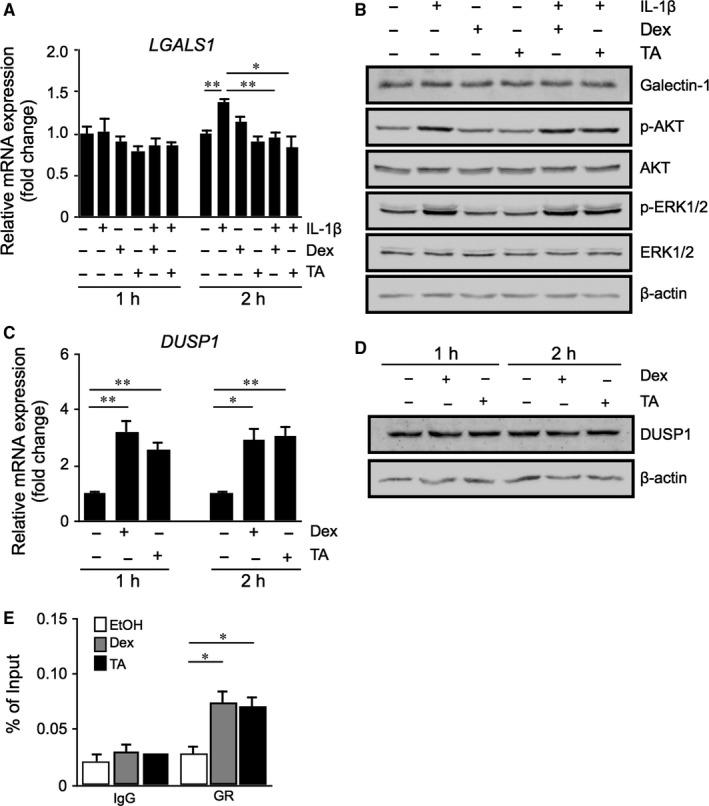
DUSP1‐independent down‐regulation of *LGALS1* expression because of deactivation of AP‐1 signalling via GR binding to AP‐1 in Müller glial cells. A, Müller glial cells were applied with dexamethasone (Dex, 1 μmol/L) or triamcinolone acetonide (TA, 1 μmol/L) and 10 ng/mL IL‐1β for 1 or 2 h, and *LGALS1* gene expression levels were analysed. B, Müller glial cells were applied with Dex (1 μmol/L) or TA (1 μmol/L) and 10 ng/mL IL‐1β for 2 h, and protein levels of galectin‐1 and phosphorylated and total forms of AKT and ERK1/2 were analysed. (C,D) Müller glial cells were treated with Dex (1 μmol/L) or TA (1 μmol/L) for 1 or 2 h, and *DUSP1* mRNA (C) and protein (D) expression levels were analysed. E, Müller glial cells were applied with Dex (1 μmol/L) or TA (1 μmol/L) and 10 ng/mL IL‐1β for 1 h, and binding of activated GR to the AP‐1/AP‐1 site complex was analysed by ChIP‐qPCR. *P < .05, **P < .01, n = 4‐6 per group

Ligand‐bound GR transactivates gene expression by binding to specific DNA sequences (ie GREs) in the promoter regions of glucocorticoid‐regulated genes such as *DUSP1* (Figure 3). In contrast to transactivation pathways mediated via GREs, transrepression processes involve the protein‐protein interaction of activated GR with transcription factors, such as AP‐1 and NF‐κB, preventing them from inducing the expression of their target genes.^19^, ^20^ Next, we investigated whether transrepression due to GR‐AP‐1 binding affects *LGALS1* gene expression in Müller glial cells. Stimulation of IL‐1β to cultured cells increased *LGALS1* mRNA expression at 2 hours, which was suppressed by treatment with dexamethasone and triamcinolone acetonide (Figure 4A); however, IL‐1β‐induced AKT and ERK1/2 phosphorylation at 2 hours were not suppressed with the glucocorticoids (Figure 4B), unlike DUSP1‐dependent dephosphorylation observed at 24 hours (Figure 3D). Consistently, administration of the glucocorticoids up‐regulated the expression of *DUSP1* mRNA (Figure 4C) but not yet DUSP1 protein (Figure 4D) at 2 hours, suggesting that the glucocorticoid‐mediated inhibition of *LGALS1* mRNA expression was also (at least during the rapid phase) achieved independently of DUSP1 enzymatic activity acquired by the GRE‐mediated transactivation pathway. Given that binding of AP‐1 to AP‐1 site in the *LGALS1* enhancer region significantly increased after stimulation with IL‐1β at 1 hour (Figure 1C), we carried out ChIP‐qPCR under this condition to examine whether GR binds to the AP‐1/AP‐1 site complex in Müller glial cells. Importantly, GR interaction with the AP‐1/AP‐1 site complex significantly increased after stimulation with dexamethasone and triamcinolone acetonide (Figure 4E). In concert with IL‐1β‐induced AP‐1 phosphorylation at 1 hour (Figure 1D) and the unchanged reporter activity of AP‐1 site in the presence of ligand‐activated GR (Figure 3A), these results showed protein‐protein interaction between activated GR and phosphorylated AP‐1 bound with AP‐1 site, resulting in *LGALS1* down‐regulation (ie transrepression).

### 3.5 Glucocorticoid‐mediated suppression of diabetes‐induced galectin‐1/*Lgals1* expression with AKT/AP‐1 and ERK/AP‐1 activation in the mouse retina

**Figure 5:**
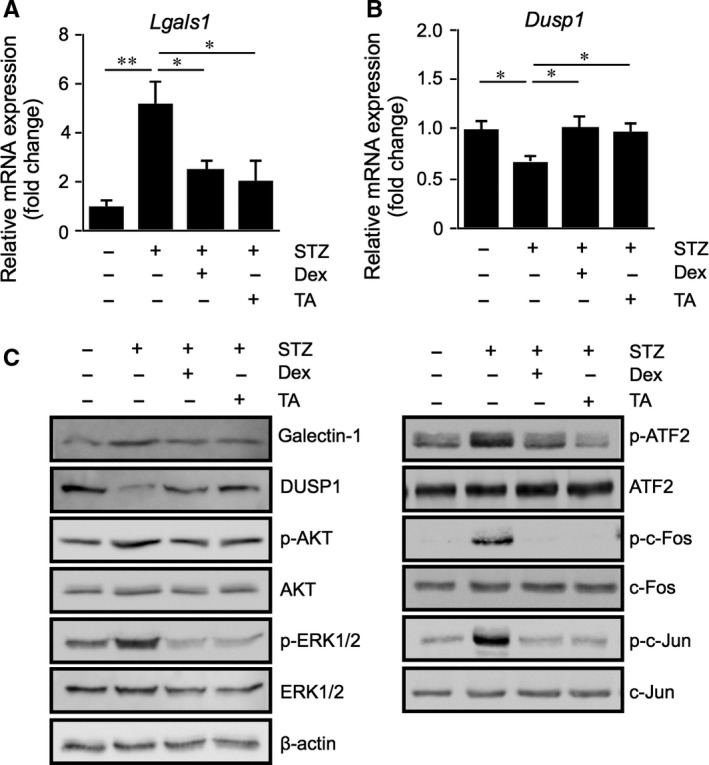
Glucocorticoid‐mediated suppression of diabetes‐induced galectin‐1/*Lgals1* expression with AKT/AP‐1 and ERK/AP‐1 activation in the mouse retina. (A‐C) Retinal *Lgals1* (A) and *Dusp1* (B) expression in mice with STZ‐induced diabetes at 2 mo. Dexamethasone (Dex, 50 pmol/eye) or triamcinolone acetonide (TA, 50 pmol/eye) were injected intravitreally to STZ mice, followed by mRNA (A,B) and protein (C) expression analyses after 24 h. *P < .05, **P < .01, n = 6‐8 per group. C, Immunoblot analyses for galectin‐1, DUSP1 and phosphorylated and total forms of AKT, ERK1/2 and AP‐1 subunits in the retina of diabetic mice treated with Dex or TA

To further confirm the in vitro suppressive effect of glucocorticoids on IL‐1β‐induced galectin‐1/*LGALS1* expression, we used the in vivo model of STZ‐induced diabetes in mice. Previously, we reported that diabetes‐induced IL‐1β was required for Müller glial expression of galectin‐1 in the retina of mice injected with STZ at 2 months.^4^, ^5^ Intravitreal injection of dexamethasone and triamcinolone acetonide significantly reduced retinal galectin‐1/*Lgals1* expression in animals with STZ‐induced diabetes at 2 months (Figure 5A,C). Additionally, diabetes‐induced reduction in *Dusp1* mRNA and protein levels was recovered by glucocorticoid treatment (Figure 5B,C). We also confirmed that treatment with dexamethasone and triamcinolone acetonide reduced diabetes‐induced phosphorylation of AKT, ERK1/2, ATF2, c‐Fos and c‐Jun as well as galectin‐1 protein levels (Figure 5C). These results suggested that induction of diabetes activated AKT/AP‐1 and ERK/AP‐1 pathways followed by retinal galectin‐1 up‐regulation, all of which were suppressed by glucocorticoid‐transactivated DUSP1.

## 4 DISCUSSION

**Figure 6:**
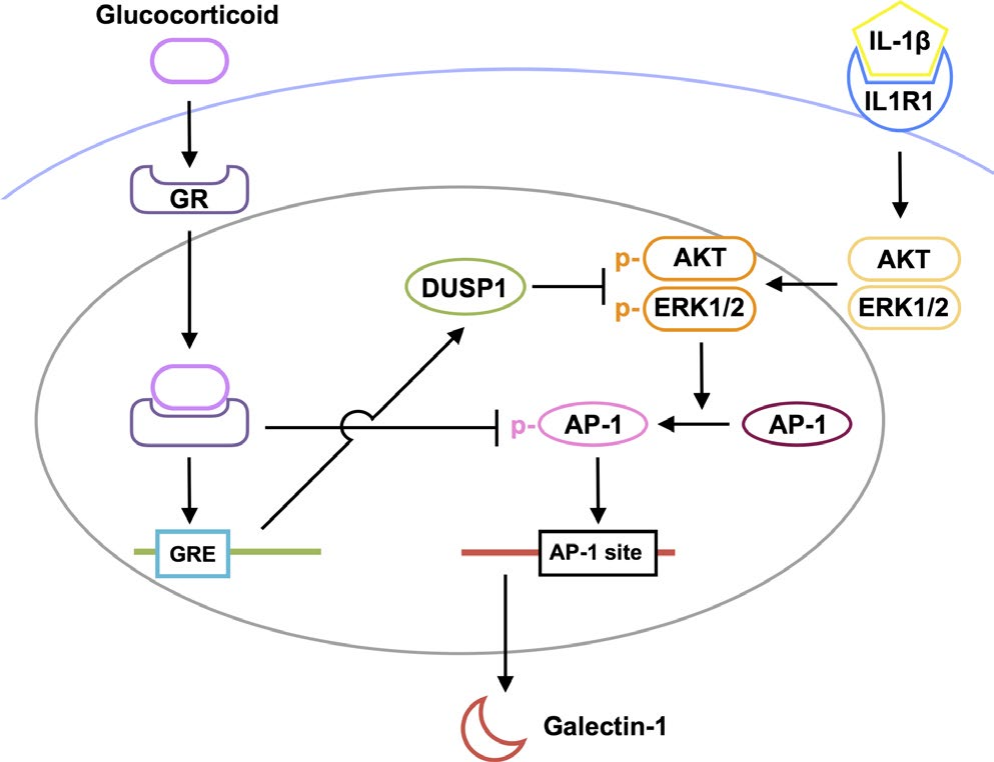
A schema showing the inhibitory effects of ligand‐bound GR on IL‐1β‐induced galectin‐1 expression with AP‐1 signalling in Müller glial cells. Ligand‐bound GR inhibits Müller glial galectin‐1 expression via DUSP1‐dependent (transactivation) and ‐independent (transrepression) deactivation of AKT/AP‐1 and ERK/AP‐1 signal transduction induced by IL‐1β

The present study is the first to show several important data on GR‐mediated inhibitory mechanisms for diabetes‐related retinal galectin‐1 expression in vitro and in vivo. AP‐1 site in the *LGALS1* enhancer region was shown to be essential for IL‐1β‐induced galectin‐1/*LGALS1* expression in Müller glial cells via AKT‐ and ERK1/2‐dependent AP‐1 phosphorylation (Figure 1). Glucocorticoids dexamethasone and triamcinolone acetonide attenuated IL‐1β‐induced galectin‐1/*LGALS1* expression by reducing the activation of AKT/AP‐1 and ERK/AP‐1 pathways in Müller glial cells (Figure 2). Ligand‐activated GR induced DUSP1 expression via GRE, leading to suppression of IL‐1β‐induced galectin‐1/*LGALS1* expression as well as AKT and ERK1/2 activation in Müller glial cells (Figure 3). Moreover, activated GR also caused DUSP1‐independent down‐regulation of IL‐1β‐induced *LGALS1* expression because of deactivation of AP‐1 signalling via GR binding to AP‐1 in Müller glial cells (Figure 4). In vivo administration of glucocorticoids to mice attenuated diabetes‐induced retinal galectin‐1/*Lgals1* expression together with AKT/AP‐1 and ERK/AP‐1 pathways (Figure 5). Supporting these in vitro and in vivo findings, immunofluorescence analyses showed co‐localization of galectin‐1 with phosphorylated AP‐1 (Figure 1) and GR (Figure 3) in GFAP‐ and DUSP1‐positive glial cells in fibrovascular tissues collected from proliferative DR patients. Our present data demonstrated the inhibitory effects of glucocorticoids on IL‐1β‐induced galectin‐1 expression via DUSP1‐dependent and ‐independent deactivation of AP‐1 signalling (ie GR‐mediated transactivation and transrepression, respectively) in Müller glial cells (Figure 6).

Previous reports disclosed that there are three key transcriptional activity sites for galectin‐1/*LGALS1* expression: (a) a region spanning the initial transcription start site (−50 bp/+50 bp) in the promoter region of the *LGALS1* gene,^11^ (b) two hypoxia‐responsive elements for hypoxia‐inducible factor‐1 binding at −441 bp to −423 bp upstream of the transcription start site^16^ and (c) an AP‐1‐driven enhancer site located at 1.5 kb downstream from the transcription start site.^12^ AP‐1 site in the *LGALS1* enhancer region was reported to play an important role in galectin‐1/*LGALS1* expression in classical Hodgkin's lymphoma and post‐transplant lymphoproliferative disorders.^12^, ^13^ Lymphoblastoid B‐cell lines highly expressed the phosphorylated AP‐1 components, which were inhibited by PI3K inhibitor,^13^ suggesting the significant involvement of PI3K/AKT signalling in AP‐1‐mediated galectin‐1/*LGALS1* expression under pathological conditions. The present study revealed that IL‐1β‐induced galectin‐1/*LGALS1* expression in Müller glial cells was caused by ERK1/2 MAPK‐ and PI3K/AKT‐mediated phosphorylation of AP‐1 subunits ATF2, c‐Fos and c‐Jun (Figure 1). In consistence with our current findings, AKT and ERK1/2 activation was previously shown to develop in the diabetic retina,^21^, ^22^ which also expressed both galectin‐1 and IL‐1β.^4^, ^5^ Taken together, galectin‐1/*LGALS1* expression in Müller glial cells proved to be regulated by AP‐1‐driven enhancer following diabetes‐related inflammatory signalling.

Glucocorticoids have a broad spectrum of functions and play important roles in the physiological regulation of a variety of processes including inflammation, immunity, metabolism and development.^23^ Glucocorticoids stimulate hormone action via binding to their receptor and transcription factor GR, which resides in the cytoplasm, leading to translocation of GR into the nucleus and modulation of gene expression in a variety of ways. The most classical model is transactivation, in which ligand‐activated GR binds to the DNA sequence called GRE in the promoter region of a target gene and up‐regulates its transcription.^19^ DUSP1, a glucocorticoid‐transactivated phosphatase localized in the nucleus, exerts anti‐inflammatory function through dephosphorylation of activated signalling molecules such as ERK1/2 and p38 MAPKs and AKT.^18^, ^24^, ^25^, ^26^ Given that *Dusp1*‐deficient mice exhibited enhanced inflammatory response and energy expenditure,^27^, ^28^, ^29^ DUSP1 is an essential component for maintaining intracellular homoeostasis that controls the threshold and magnitude of signal transduction. In consistence with our human tissue data (Figure 3), DUSP1 was reported to exist in rat retinal cells including Müller glial cells^30^ and work as a suppressive regulator of inflammation in bovine Müller glial cells.^31^ Indeed, we revealed that dexamethasone and triamcinolone acetonide induced DUSP1 expression together with deactivation of AKT/AP‐1 and ERK/AP‐1 signalling pathways (Figure 2), whereas silencing *DUSP1* reversed the glucocorticoid‐mediated inhibition of AKT and ERK1/2 phosphorylation (Figure 3), further confirming the role of DUSP1 in human Müller glial cells. In accordance with these in vitro findings, intravitreal administration of glucocorticoids to diabetic mice up‐regulated DUSP1 to switch off the activated AKT/AP‐1 and ERK/AP‐1 signalling pathways, leading to the reduction of retinal galectin‐1/*Lgals1* expression (Figure 5). Retinal DUSP‐1 down‐regulation currently seen in diabetic mice was comparable with previous reports showing myocardial and hippocampal DUSP‐1 decreases in rats with STZ‐induced diabetes.^32^, ^33^ Diabetes‐induced reduction of DUSP‐1 in vivo would theoretically be a common pathogenic mechanism without organ specificity leading to systemic chronic inflammation, given its in vitro response to high glucose (ie down‐regulation) in vascular smooth muscle cells.^34^ However, we could not exclude the possible involvement of other anti‐inflammatory pathways in glucocorticoid‐mediated suppression of diabetes‐induced galectin‐1 up‐regulation, because we also found that inhibition of hypoxia‐induced *LGALS1* expression was not mediated by DUSP1 in Müller cells treated with dexamethasone and triamcinolone acetonide (unpublished data).

Alternatively, activated GR suppresses inflammatory gene transcription by interacting with transcription factors (ie direct protein‐protein binding) in the regulation of transcriptional response to glucocorticoids, which is referred to as transrepression.^19^ In consistence with our ChIP‐qPCR data (Figure 4), GR can be tethered via DNA‐bound transcription factors to the DNA sequences (eg AP‐1 site) that do not contain GR’s recognition site.^35^ This regulatory mechanism of action involves the interaction between activated GR and inflammation‐related transcription factors including AP‐1, NF‐κB and STAT (signal transduction and transcription proteins),^20^ allowing GR to down‐regulate the expression of their downstream molecules such as metalloproteinases.^36^ Glucocorticoids are known to rapidly increase the transcription of *DUSP1* in lung adenocarcinoma cells,^37^ consistent with our data on Müller glial cells (Figure 4); however, it was additionally found that glucocorticoids suppressed IL‐1β‐induced *LGALS1* gene expression even before phosphorylated AKT and ERK1/2 protein levels did not decrease (ie DUSP1 protein did not increase). During the rapid phase, it was theorized from these results that ligand‐activated GR interacted with phosphorylated AP‐1 bound with AP‐1 site in the *LGALS1* gene, leading to DUSP1‐independent repression of *LGALS1* transcription in Müller glial cells.

Once the DUSP1 transactivation process is completed, however, this transrepression pathway is likely to be ceased from DUSP1‐mediated lack of phosphorylated AP‐1. This is consistent with our current data that glucocorticoid‐mediated suppression of galectin‐1 at 24 hours was almost completely dependent on DUSP‐1 induced in Müller glial cells (Figure 3). Similarly, our in vivo results on retinal galectin‐1 at 24 hours after glucocorticoid application were thought to stem from DUSP1 transactivation and subsequent dephosphorylation of AKT, ERK1/2 and AP‐1 signalling molecules (Figure 5). Transrepression would therefore be regarded as an anti‐inflammatory action more rapidly exerted until transactivation is instead established. This is supported by our frequent observations in clinical practice that diabetic macular oedema resolves within a few hours after glucocorticoid injection to the eye.^38^, ^39^ The much faster effect of glucocorticoid drugs than anti‐VEGF agents is also attributable in part to GR‐unrelated non‐genomic mechanisms (ie non‐specific interactions of glucocorticoids with cellular membranes).^20^ Nevertheless, GR‐mediated transrepression is theorized to be faster in efficacy than transactivation by at least several hours of duration needed for acquiring the MAPK phosphatase activity, which was actually true of glucocorticoid‐induced down‐regulation of galectin‐1 in Müller glial cells (Figures 3 and 4). Our present data demonstrated the inhibitory effects of glucocorticoids on retinal glial galectin‐1 expression via DUSP1‐dependent and ‐independent deactivation of AP‐1 signalling (ie GR‐mediated transactivation and transrepression, respectively), highlighting therapeutic implications for the management of DR, especially if patients suffer from vision‐threatening macular oedema.

## CONFLICT OF INTEREST

The authors declare no competing financial interests.

## AUTHOR CONTRIBUTION

AK designed research; IH, AK and KN performed the experiments; IH and AK analysed the data; AK and SI wrote the paper; and all authors approved the final version submitted for publication.

## Data Availability

All data generated or analysed during this study are included in this published article and its supplementary information files.
