# Supplementary material for: Glucocorticoid receptor inhibits Müller glial galectin‐1 expression via DUSP1‐dependent and ‐independent deactivation of AP‐1 signalling
Source: J Cell Mol Med. 2019 Jul 21;23(10):6785–96. doi: 10.1111/jcmm.14559 (PMC6787449; doi:10.1111/jcmm.14559)
Supplement: Supplementary file 1 [file JCMM-23-6785-s001.pdf]

Supplementary Figure S1

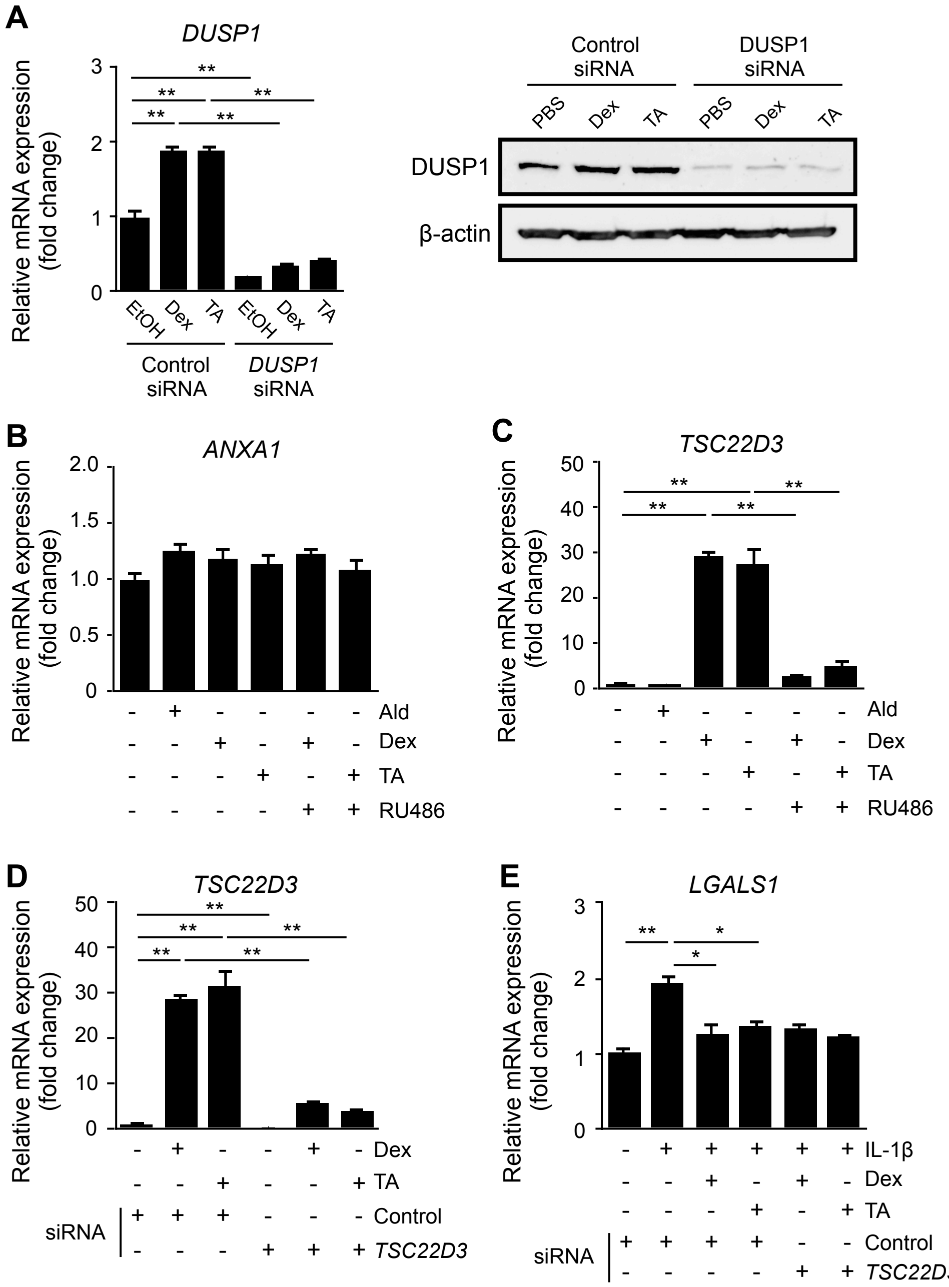

Supplementary Figure S1. GR-transactivated anti-MAPK molecules as candidate regulators for glucocorticoid-mediated suppression of IL-1β-induced galectin-1/*LGALS1* expression in Müller glial cells

(A) *DUSP1* mRNA and protein expression levels in *DUSP-1*-knockdown Müller glial cells treated with dexamethasone (Dex, 1 μM) or triamcinolone acetonide (TA, 1 μM) for 24 hours. n = 4-6 per group. (B, C) Müller glial cells were pretreated with RU486 (1 μM) for 30 minutes before application with aldosterone (Ald, 1 μM), Dex (1 μM) or TA (1 μM) for 24 hours, and *ANXA1* and *TSC22D3* gene expression levels were analyzed. n = 6 per group. (D) *TSC22D3* mRNA and protein expression levels in *TSC22D3*-knockdown Müller glial cells treated with Dex (1 μM) or TA (1 μM) for 24 hours. (E) After application to *TSC22D3*-knockdown cells with Dex (1 μM) or TA (1 μM) and 10 ng/ml IL-1β for 24h, *LGALS1* gene expression levels were analyzed. *$p < 0.05$, **$p < 0.01$, n = 6 per group.
