# Supplementary material for: Glucocorticoid receptor inhibits Müller glial galectin‐1 expression via DUSP1‐dependent and ‐independent deactivation of AP‐1 signalling
Source: J Cell Mol Med. 2019 Jul 21;23(10):6785–96. doi: 10.1111/jcmm.14559 (PMC6787449; doi:10.1111/jcmm.14559)
Supplement: Supplementary file 2 [file JCMM-23-6785-s002.pdf]

Supplementary Table S1. Primer sequences used in quantitative ChIP-qPCR and real-time qPCR

Quantitative ChIP-qPCR

**Target gene**	**Sequence**
AP-1-binding site in the *LGALS1* enhancer region	
	forward 5'- CCA AGC CCA CAT CTC CTC -3'
	reverse 5'- GAG GCT GCA GCT GGT TTA GT -3'
GR-binding site in the *DUSP1* promoter region	
	forward 5'- GCG CAG GCG AAA ACA CAC AA -3'
	reverse 5'- CGA CGC GCT AGG TAC ACA AAC A -3'

Real-time qPCR

**Target gene**	**Sequence**
Human	
LGALS1	forward 5'- CGC TAA GAG CTT CGT GCT GAA C -3'
	reverse 5'- CAC ACC TCT GCA ACA CTT CCA G -3'
DUSP1	forward 5'- CTG CCT TGA TCA ACG TC TCA -3'
	reverse 5'- CTG TGC CTT GTG GTT GTC CT -3'
ANXA1	forward 5'- GCG AAA CAA TGC ACA GCG TCA AC -3'
	reverse 5'- CAA CCT CCT CAA GGT GAC CTG T -3'
TSC22D3	forward 5'- ATC TGC AAC CGC AAC ATC GAC C -3'
	reverse 5'- GCA TAC ATC AGA TGA TTC TTC ACC -3'
ACTB	forward 5'- CTG GAA CGG TGA AGG TGA CA -3'
	reverse 5'- AAG GGA CTT CCT GTA ACA ATG CA -3'
Mouse	
Lgals1	forward 5'- GTC TCA GGA ATC TCT TCG CTT C -3'
	reverse 5'- TCC CCG AAC TTT GAG ACA TTC -3'
	probe 5'- TTC AAT CAT GGC CTG TGG TCT GGT- 3'
Dusp1	forward 5'- GGC CAG CTG CTG CAG TTT GAG T -3'
	reverse 5'- AGG TGC CCC GGT CAA GGA CA -3'
Actb	forward 5'- CAT CCG TAA AGA CCT CTA TGC CAA C -3'
	reverse 5'- ATG GAG CCA CCG ATC CAC A -3'
